# *Garcinia cambogia* Ameliorates Non-Alcoholic Fatty Liver Disease by Inhibiting Oxidative Stress-Mediated Steatosis and Apoptosis through NRF2-ARE Activation

**DOI:** 10.3390/antiox10081226

**Published:** 2021-07-29

**Authors:** Joo-Hui Han, Min-Ho Park, Chang-Seon Myung

**Affiliations:** 1 Department of Pharmacology, College of Pharmacy, Chungnam National University, Daejeon 34134, Korea; 2 Institute of Drug Research and Development, College of Pharmacy, Chungnam National University, Daejeon 34134, Korea; minho.park.cnu@gmail.com

**Keywords:** *Garcinia cambogia*, non-alcoholic fatty liver disease (NAFLD), steatosis, apoptosis, reactive oxygen species, antioxidant, NRF2, hydroxycitric acid

## Abstract

Excessive free fatty acids (FFAs) causes reactive oxygen species (ROS) generation and non-alcoholic fatty liver disease (NAFLD) development. *Garcinia cambogia* (*G. cambogia*) is used as an anti-obesity supplement, and its protective potential against NAFLD has been investigated. This study aims to present the therapeutic effects of *G. cambogia* on NAFLD and reveal underlying mechanisms. High-fat diet (HFD)-fed mice were administered *G. cambogia* for eight weeks, and steatosis, apoptosis, and biochemical parameters were examined in vivo. FFA-induced HepG2 cells were treated with *G. cambogia*, and lipid accumulation, apoptosis, ROS level, and signal alterations were examined. The results showed that *G. cambogia* inhibited HFD-induced steatosis and apoptosis and abrogated abnormalities in serum chemistry. *G. cambogia* increased in NRF2 nuclear expression and activated antioxidant responsive element (ARE), causing induction of antioxidant gene expression. NRF2 activation inhibited FFA-induced ROS production, which suppressed lipogenic transcription factors, C/EBPα and PPARγ. Moreover, the ability of *G. cambogia* to inhibit ROS production suppressed apoptosis by normalizing the Bcl-2/BAX ratio and PARP cleavage. Lastly, these therapeutic effects of *G. cambogia* were due to hydroxycitric acid (HCA). These findings provide new insight into the mechanism by which *G. cambogia* regulates NAFLD progression.

## 1. Introduction

Obesity is a chronic disease resulting from an energy imbalance caused by hypercaloric food intake and insufficient energy expenditure [1] and is strongly associated with type 2 diabetes, hyperlipidemia, and non-alcoholic fatty liver disease (NAFLD) [2]. NAFLD is the most common chronic liver disease in western societies and is well recognized as the hepatic manifestation of metabolic disease [3]. NAFLD is characterized by excessive fat accumulation in hepatocytes and is strongly associated with obesity and energy imbalance [4]. If this pathological state is not properly treated, it can progress to non-alcoholic steatohepatitis (NASH) and continue to progress to cirrhosis and ultimately to hepatocellular carcinoma [5].

NAFLD is a various factorial disease associated with genetic, epigenetic, and environmental factors [6]. Although its pathogenesis is still not entirely clear, the “two-hit hypothesis” proposes an explanation for the development of NAFLD [7]. The first hit results from insulin resistance, which causes an imbalance between hepatic lipid influx and clearance [8]. The second hit involves the perturbation of redox homeostasis, causing reactive oxygen species (ROS) generation. Redox imbalance leads to hepatic mitochondrial dysfunction and inflammatory and oxidative processes [9]. An updated theory is the “multiple-hit” hypothesis, which proposes the involvement of a number of factors, including insulin resistance, inflammation, and oxidative stress [10]. One of the factors that contribute to the “multiple-hit” is oxidative stress, which is considered the main contributor to the progression of NAFLD [11]. Oxidative stress is an important cause of NAFLD, and regulation of ROS generation is one important therapeutic strategy.

Excessive accumulation of free fatty acids (FFAs) due to the intake of high-calorie food induces ROS production, and abnormal levels of ROS can mediate the progression of NAFLD, causing a breakdown of lipid homeostasis via upregulation of lipogenic genes [12,13,14]. In addition, ROS can cause apoptosis of hepatocytes and increase liver damage to promote the progression of NAFLD by inducing mitochondrial dysfunction [12]. Thus, several studies have reported that antioxidants have therapeutic effects on NAFLD by scavenging ROS and inhibiting de novo lipogenesis and apoptosis via regulating related signaling pathways [15,16,17]. Phloroglucinol, a phenolic compound of natural origin, attenuates palmitic acid and hydrogen peroxide-induced oxidative damage and reduced steatosis in HepG2 cells via strengthening the enzymatic and nonenzymatic antioxidants barriers [18]. Berberine, an isoquinoline quaternary alkaloid majorly extracted from a Chinese herb named *Coptis chinensis*, ameliorates non-alcoholic hepatic steatosis via reducing nicotinamide adenine dinucleotide phosphate oxidase 2 (Nox2)-dependent cytoplasmic ROS production and mitochondrial ROS production [19].

Normal cells can maintain oxidative homeostasis due to the operation of antioxidant defense systems [20]. Nuclear factor erythroid 2-related factor 2 (NRF2), which is a transcription factor and binds to the antioxidant response element (ARE), activates cellular antioxidant response by inducing the transcription of antioxidant genes, such as heme oxygenase 1 (*HMOX1*) and superoxide dismutase (*SOD*) to regulate oxidative stress [21]. Notably, NRF2 has been demonstrated to contribute to the suppression of NAFLD by negative regulation of lipogenic gene expression [22]. Furthermore, NRF2 overexpression increased antiapoptotic mitochondrial protein Bcl-2, implying attenuation of oxidative and apoptotic response [23]. Therefore, the regulation of NRF2 not only controls oxidative stress but also ameliorates NAFLD development, which is a potential therapeutic target to protect from liver disease.

*Garcinia cambogia* (*G. cambogia*) is a fruit that is native to Southeast Asia and is currently used as a weight-loss supplement [24]. There are many studies suggesting the anti-obesity effect of *G. cambogia* mediated by an increase in fat oxidation [25], a decrease in de novo lipogenesis [26], and inhibition of mitotic clonal expansion (MCE) in the early stage of adipocyte differentiation [27]. Interestingly, *G. cambogia* reduced liver weight gain, vacuolization, and lipid droplet numbers in the liver tissues of high-fat diet (HFD)-fed mice, implying a therapeutic effect on NAFLD [28,29]. Despite these findings, the underlying mechanisms and interactions of signaling mediators by which *G. cambogia* inhibits NAFLD remains poorly understood.

In this study, we investigated the therapeutic effects of *G. cambogia* on HFD-fed liver tissues and FFA-treated HepG2 cells. Our findings show the inhibitory effect of *G. cambogia* on NAFLD through suppressing HFD- and FFA-induced steatosis and apoptosis. These effects were due to an increase of NRF2-mediated attenuation of hepatic lipogenic gene transcription and apoptosis-related signal transduction.

## 2. Materials and Methods

### 2.1. Reagents, Chemicals, and Antibodies

Dulbecco’s modified Eagle’s medium (DMEM), fetal bovine serum (FBS), phosphate-buffered saline (PBS), penicillin/streptomycin, and trypsin-ethylenediaminetetraacetic acid (EDTA) were obtained from Gibco, Inc. (Thermo Fisher Scientific, Waltham, MA, USA). Sodium oleate, sodium palmitate, oil red O, isopropanol, (−)-epigallocatechin gallate (EGCG), 3-(4,5-dimethylthiazol-2-yl)-2,5-diphenyltetrazolium bromide (MTT), digitonin, N-acetyl-L-cysteine (NAC), anti-rabbit-fluorescein isothiocyanate (FITC, F0382), and potassium hydroxycitrate tribasic monohydrate were purchased from Sigma-Aldrich (St. Louis, MO, USA). Bovine serum albumin was obtained from Bovogen Biologicals (Melbourne, Australia). Anti-C/EBPα (8178), anti-PPARγ (2443), anti-BAX (2772), anti-PARP (9532), anti-NRF2 (12721), anti-Lamin A/C (4777), and goat anti-rabbit (7074) antibodies were purchased from Cell Signaling Technology (Beverly, MA, USA). Anti-Bcl-2 (sc-7382) antibody was obtained from Santa Cruz Biotechnology, Inc. (Dallas, TX, USA). Anti-β-actin (LF-PA0207) and goat anti-mouse (LF-SA8001) antibodies were purchased from Abfrontier (Geumcheon, Seoul, Korea). *Garcinia cambogia* (*G. cambogia*) powder (main component: hydroxycitric acid, 59.55%) was obtained from Mirae Biotech, Inc. (Pocheon, Gyeonggi-do, Korea). All other chemicals were of analytical grade.

### 2.2. Animals and Diet

Four-week-old male C57BL/6N mice (average weight, 22 g) were obtained from Orient Bio Inc. (Seongnam, Korea). The animals were provided normal chow and water ad libitum in the animal facility under controlled temperature (22 ± 2 °C), humidity (50 ± 5%), and lighting (12/12 h dark/light cycle, lights on at 7:00 a.m.) conditions. After a 1-week acclimatization period, animal experiments were performed by minimizing the use of the animals and their discomfort according to procedures approved by the Institutional Animal Care and Use Committee of Chungnam National University (2019012A-CNU-190) and the Animal Research: Reporting of In Vivo Experiments (ARRIVE) guidelines [30].

To induce obesity, mice were randomly divided into five groups (*n* = 7 per group): normal diet (ND, control), high-fat diet (HFD), HFD + 200 mg/kg *G. cambogia* (HFD + Ga 200), HFD + 400 mg/kg *G. cambogia* (HFD + Ga 400) and HFD + 20 mg/kg orlistat (HFD + Orli 20, positive control of anti-obesity [31] and anti-steatosis [32,33]). The control group mice were fed an ND (10% kcal fat, 20% kcal protein, 70% kcal carbohydrate, 3.82 kcal/g, R12450B, Research Diets, New Brunswick, NJ, USA), and the HFD group mice were fed a high-fat diet (40% kcal fat, 17% kcal protein, 43% carbohydrate, 4.67 kcal/g, R12079B, Research Diets) ad libitum with free access to water for 8 weeks. Mice were administered *G. cambogia* or orlistat dissolved in 0.5% carboxymethyl cellulose (CMC) orally every day. The weight gains from initial and final body weights are shown in Table 1.

### 2.3. Dosage Information

The dosages of *G. cambogia* were determined by consideration of the equivalent conversion of human dose [34] for a mouse model [35] and several studies, including an animal model [36,37]. The dosages of *G. cambogia* used for weight loss range from 1667 to 4668 mg (taken in divided doses) daily for 60 kg humans [34], and the *G. cambogia* dose for a mouse model based on a conversion of these dose regiment ranges from 307.5 to 947.1 mg/kg daily [35]. Considering dosages of other studies (200–250 mg/kg) [36,37], low dose (200 mg/kg) and high dose (400 mg/kg) were determined. In addition, the dosage of orlistat was determined similar to *G. cambogia*. The dosages of orlistat used for weight loss range from 180 to 360 mg (taken in divided doses) daily for 60 kg humans [38], and the orlistat dose for a mouse model based on a conversion of these dose regiments ranges from 32.2 to 64.4 mg/kg daily. Considering dosages of other studies (10 mg/kg) [39], orlistat dosage (20 mg/kg) was determined.

### 2.4. Histological Analysis

At the end of the experimental period, all animals were fasted overnight and anesthetized by CO_2_ administration. After the mice were euthanized by cervical dislocation, liver, heart, spleen, lung, and kidney tissues were collected, weighed, rinsed, fixed with 4% paraformaldehyde, and embedded in paraffin. The tissues were cut into 4-μm-thick sections. The sections were stained with hematoxylin and eosin (H&E) for histological examination, and images were acquired using a microscope (Nikon Eclipse Ti, Nikon Instruments Inc., Tokyo, Japan). The scale bars in the images represent 50 μm. The degree of steatosis was quantified as described previously [40]. Briefly, macrovesicular steatosis (vacuoles displaced the nucleus to the side) and microvesicular steatosis (vacuoles not displaced around the nucleus) were both separately scored, and the severity was graded based on the percentage of the total area affected. The scores were: 0 (<5), 1 (5–33%), 2 (33–66%) and 3 (66%) [41]. Five fields were analyzed per animal, and an average score of each animal was considered for statistical analysis. In addition, the liver index was calculated as follows:

Liver index = liver weight (g)/body weight (g)

In vivo studies were performed in a blinded fashion at the histological analysis stage. For the quantification of tissue staining and pathological evaluation of animal specimens, the investigator was blinded to ensure an unbiased interpretation of the results.

### 2.5. Serum Analysis

At the end of the experimental period, blood samples were collected from the abdominal aorta and centrifuged at 6000 rpm for 20 min to separate the serum. The levels of alanine aminotransferase (ALT), aspartate aminotransferase (AST), triglycerides, and total cholesterol were analyzed using a DRI-CHEM 7000i (Fujifilm, Tokyo, Japan). The malondialdehyde (MDA) content was determined using an OxiSelect™ TBARS Assay Kit (MDA Quantitation) (Cell Biolabs Inc., San Diego, CA, USA) following the manufacturer’s protocol. The levels of MDA were measured at 532 nm using a microplate reader (Tecan Group Ltd., Männedorf, Switzerland). 

### 2.6. Cell Culture

Human hepatoblastoma HepG2 cells were obtained from the American Type Culture Collection (Cat. No. ATCC HB-8065, Manassas, VA, USA) and grown in DMEM supplemented with 10% (*v*/*v*) heat-inactivated fetal bovine serum, 100 IU/mL penicillin, and 100 μg/mL streptomycin at 37 °C in a humidified incubator under 95% air and 5% CO_2_. Cells were seeded on different types of plates and serum-starved for 24 h in a serum-free medium before experimental treatment. *G. cambogia* extract (dissolved in DW) and other reagents (dissolved in DMSO to a final DMSO concentration in the medium of ≤0.2%) were added for the indicated time periods. HepG2 cells were used at passages 4–7.

### 2.7. Free Fatty Acids (FFAs) Solution Preparation

The FFAs solution was prepared by previously described methods [42]. Briefly, the FFA-containing stock solution was prepared by conjugation of oleate (0.67 mM) and palmitate (0.33 mM) with 1% FFA-free BSA in a serum-free medium (final FFA concentration, 1 mM). After 24 h of incubation with FFA-containing medium and experimental treatments, the extent of steatosis, apoptosis, ROS levels, and protein expression levels was evaluated.

### 2.8. MTT Assay

Cell viability was assessed using the MTT assay. HepG2 cells were seeded on 96-well plates and incubated in a serum-free medium. After 24 h, the *G. cambogia*, other reagents, and 100 μg/mL digitonin (used as a positive control for cytotoxicity [43]) were added for 24 h. After the reaction was terminated, 5 mg/mL of MTT reagent was added, and the cells were incubated at 37 °C under humidified 5% CO_2_. After 3 h, dimethyl sulfoxide was added to dissolve any formed crystals. The absorbance was measured at 565 nm using a microplate reader (Tecan Group Ltd.). All results are expressed as fold changes relative to the control (no stimulation) value.

### 2.9. Oil Red O Staining

Lipid droplets in cells were examined using oil red O staining after the reaction was terminated. HepG2 cells were fixed using 4% formaldehyde for 1 h and washed with 60% isopropanol. Then, the fixed cells were stained with filtered oil red O solution in 60% isopropanol for 10 min. After the oil red O solution was removed, the stained cells were washed with distilled water. Images of lipid droplets in the stained cells were acquired using an Olympus IX71 digital microscope (Olympus, Tokyo, Japan). The stained lipid droplets were dissolved in 100% isopropanol and quantified by measuring the absorbance at 520 nm using a microplate reader (Tecan Group Ltd.).

### 2.10. Western Blot Analysis

Western blotting was performed to detect protein levels in cells and tissues. Proteins were extracted in ice-cold RIPA buffer (50 mM Tris-HCl, pH 8.0, 150 mM NaCl, 1.0% NP-40, 2 mM EDTA, 5 mM NaF, 1 mM phenylmethylsulfonyl fluoride [PMSF], 1 mM sodium orthovanadate, 0.5% sodium deoxycholate, and 0.1% sodium dodecyl sulfate [SDS]). The protein concentration in the lysates was quantified using a BCA protein assay kit (Pierce, Rockford, IL, USA). Equivalent amounts of protein were separated using sodium dodecyl sulfate-polyacrylamide gel electrophoresis (SDS-PAGE, 7.5–12.5%) and transferred to polyvinylidene fluoride (PVDF) membranes (ATTO Corp., Tokyo, Japan). The membranes were blocked with TBS-T (10 mM Tris, 150 mM NaCl, and 0.1% Tween-20, pH 7.6) containing 5% bovine serum albumin (BSA) for 1 h. Next, the membranes were incubated with primary antibodies overnight and were then incubated with secondary antibodies for 6 h at 4 °C. Specific signals were detected using an enhanced chemiluminescence reagent (ATTO Corp., Tokyo, Japan). The band densities were quantified using Image Lab software (Version 5.2.1, Bio-Rad, Hercules, CA, USA).

### 2.11. Quantitative Real-Time PCR (qPCR)

Total RNAs in cells and tissues were isolated using TRIzol™ (Invitrogen) according to the manufacturer’s instructions. Reverse transcription was performed using AccuPower CycleScript RT Premix (Bioneer, Daejeon, Korea) and 1 µg RNA in a thermocycler. Real-time PCR amplification was performed using 96-well optical plates, with TOPreal™ qPCR 2X PreMIX (SYBR Green with low ROX) (Enzynomics, Daejeon, Korea), 400 nM each of forward and reverse primer and 1 µL of cDNA and H_2_O in a 20 µL reaction volume. Real-time fluorescence of PCR products was detected using CFX96 Real-Time Detection System (Bio-Rad, Hercules, CA, USA) at following thermocycling conditions: 1 cycle of 95 °C for 3 min; 39 cycles of 95 °C for 10 s, and 60 °C for 30 s; 1 cycle of 95 °C for 10 s and 65 °C for 5 s followed by 0.5 °C increments at 5 s/step back to 95 °C. Only primer pairs leading to the synthesis of a single fragment with the appropriate size were used in this study. Primer sets used for this study were listed in Table S1. The relative gene expression was calculated using a 2^−∆∆ct^ method, which was normalized by *β-actin* expression level.

### 2.12. TUNEL Staining

A Click-iT™ Plus TUNEL Assay for in situ apoptosis detection (Invitrogen, Carlsbad, CA, USA) was used for TUNEL staining, which was performed using sections from liver tissues. As positive controls, sections were pre-treated with DNase I (30 U). Images were acquired using a confocal laser microscope (K1-Fluo, Nanoscope Systems, Daejeon, Korea) using K1-image software (Nanoscope Systems, Daejeon, Korea). The number of TUNEL-positive cells was quantified using ImageJ software (Version 1.53c, NIH, Bethesda, MD, USA).

### 2.13. Apoptosis Detection Assay

Apoptosis-mediated cell death was analyzed by annexin V (AV) and propidium iodide (PI) staining using a FITC Annexin V Apoptosis Detection Kit I (BD Pharmingen™, San Jose, CA, USA) according to the manufacturer’s guidelines. Briefly, cells were collected by trypsinization and centrifuged at 5000 rpm for 3 min. The pellets were washed carefully with PBS and centrifuged. Then the cells were stained with AV-FITC/PI solution. The extent of AV-FITC and PI staining was measured using FACS Canto II and BD FACS Diva software. AV^−^ PI^−^ (viable), AV^+^ PI^−^ (early apoptotic), AV^+^ PI^+^ (late apoptotic), and AV^−^ PI^+^ (necrotic) cells were analyzed using FlowJo software (Version 10, FlowJo LLC, Ashland, OR, USA), and the data are summarized in one graph.

### 2.14. Caspase-3 Activity

The in vitro and in vivo caspase-3 activity assay was carried out in accordance with the instructions provided by the Caspase-3 Assay Kit (Abcam, MA, USA). Briefly, lysates of cells or liver tissues were collected, and reaction buffer with dithiothreitol (DTT, 10 mM) was added. Then, the caspase-3 substrate in the kit (DEVD-*p*-NA, 200 μM) was treated for 60 min at 37 °C. The absorbance was measured at 405 nm using a microplate reader (Tecan Group Ltd.). All results are expressed as fold changes relative to the control (no stimulation) value.

### 2.15. Determination of Intracellular ROS Level

HepG2 cells were seeded into black 96-well plates. Cells incubated with FFAs were cotreated with *G. cambogia* and N-acetyl-L-cysteine (NAC, 4 mM, positive control for antioxidant activity [44]). After the reaction was terminated, the medium was removed, and H_2_DCFDA (20 μM) diluted in serum-free medium was added to the cells and incubated for 30 min at 37 °C. The cells were washed and filled with PBS, and the fluorescence intensity (excitation = 485 nm; emission = 530 nm) was measured using a fluorescence reader (Infinite F200, Tecan Group Ltd.). All results are expressed as fold changes relative to the control (no stimulation) value.

### 2.16. Cell Fractionation

Cell fractionation was performed as described previously [45]. Briefly, HepG2 cells were treated with FFA and *G. cambogia* for 12 h. After the reaction was terminated, cells were centrifuged at 800× *g* for 10 min and homogenized in buffer 1 (250 mM sucrose, 50 mM Tris-HCl, pH 7.4, 5 mM MgCl_2_, 2 mM EDTA, 5 mM NaF, 1 mM PMSF, and 1 mM sodium orthovanadate) and kept on ice for 30 min. Then, the homogenate was mixed by vortexing for 15 s and centrifuged at 800× *g* for 15 min at 4 °C to obtain supernatant (S0) and pellet (P0). The pellet (P0) was mixed with buffer 1 by vortexing for 15 s and centrifuged at 500× *g* for 15 min at 4 °C. The obtained pellet was resuspended in buffer 2 (20 mM HEPES, pH 7.9, 1.5 mM MgCl_2_, 0.5 M NaCl, 20% glycerol, 1% Triton X-100, 2 mM EDTA, 5 mM NaF, 1 mM PMSF and 1 mM sodium orthovanadate) by vortexing for 15 s and kept on ice for 30 min. Then, the homogenate was placed on ice in a Vibra-Cell sonicator (Sonics, Newtown, CT, USA) for 8 × 5 s bursts with 10 s intervals. The homogenate was centrifuged at 9000× *g* for 30 min at 4 °C, and the supernatant was resuspended in 5× SDS loading buffer to generate the nuclear fraction. Western blot analysis was performed to identify nuclear fraction proteins. Lamin A/C was used as a loading control for the nucleus.

### 2.17. Immunofluorescence

HepG2 cells were seeded with coverslips and placed in 24-well plates. Then, HepG2 cells were treated with FFA and *G. cambogia* for 12 h. After the reaction was completed, cells were fixed with 4% formaldehyde and permeabilized with 0.25% Triton X-100 in PBS and 1% BSA for 5 min. Then cells were blocked with 5% goat serum in PBS. After 1 h, cells were incubated with NRF2 primary antibodies for 16 h at 4 °C and subsequently treated with secondary antibodies in PBS with 3% BSA for 2.5 h at room temperature. Then cell nuclei were stained by 4′,6-diamidino-2-phenylindole (DAPI), and observed under a confocal laser microscope (K1-Fluo, Nanoscope systems, Daejeon, Korea) using K1-image software (Nanoscope systems, Daejeon, Korea). The intensity of nuclear NRF2 was measured by ImageJ software (Version 1.53c, NIH, Bethesda, MD, USA).

### 2.18. Dual-Luciferase Assay

HepG2 cells were co-transfected with a DNA mixture containing the pGL4.37[luc2P/ARE/Hygro] reporter (Promega, Madison, WI, USA), renilla luciferase control reporter (pRL-TK, Thermo Fisher Scientific, Waltham, MA, USA), and 1 μL of Lipofectamine 2000. After 4 h, cells were treated with FFA and *G. cambogia* for 12 h. After cells were harvested using passive lysis buffer, ARE promoter activity was measured with the Dual-Luciferase Reporter Assay System (Promega) using a Glomax 20/20 luminometer reader (Promega). Specific promoter activity was expressed as the relative activity ratio of firefly luciferase to Renilla luciferase.

### 2.19. Identification of Hydroxycitric Acid in G. cambogia Using Liquid Chromatography-High Resolution Mass Spectrometry

For the identification of hydroxycitric acid in *G. cambogia*, the content of hydroxycitric acid was analyzed using liquid chromatography-high resolution mass spectrometry (LC-HRMS). The *G. cambogia* powder was dissolved at a concentration of 1 μg/mL in distilled water. After vortexing for 1 min, the solution was centrifuged at 12,000 rpm for 7 min. The supernatant was transferred to an LC vial for LC-HRMS analysis. The LC-HRMS system consisted of a CTC HTS PAL auto-sampler (LEAP Technologies, Carrboro, NC, USA), LC-20AD pumps (Shimadzu Corporation, Columbia, MD, USA), CBM-20A controller (Shimadzu Corporation), and a hybrid quadrupole time-of-flight mass spectrometer 5600 with an electrospray ionization source (Sciex, Foster City, CA, USA). The hybrid quadrupole time-of-flight mass spectrometer 5600, equipped with an electrospray ionization source, was operated in the negative ion mode. The mass transition of parent-to-parent ion was used for hydroxycitric acid (*m*/*z* 207.0→207.0). Water containing 0.1% formic acid (A) and acetonitrile containing 0.1% formic acid (B) was used as mobile phase. The flow rate was 0.4 mL/min. Error ppm was applied for the accuracy of measurement. Data processing was performed using the Analyst^®^ TF 1.6 and MultiQuant™ software (Version 3.0.3, Sciex).

### 2.20. Statistical Analyses

**Figure 1:**
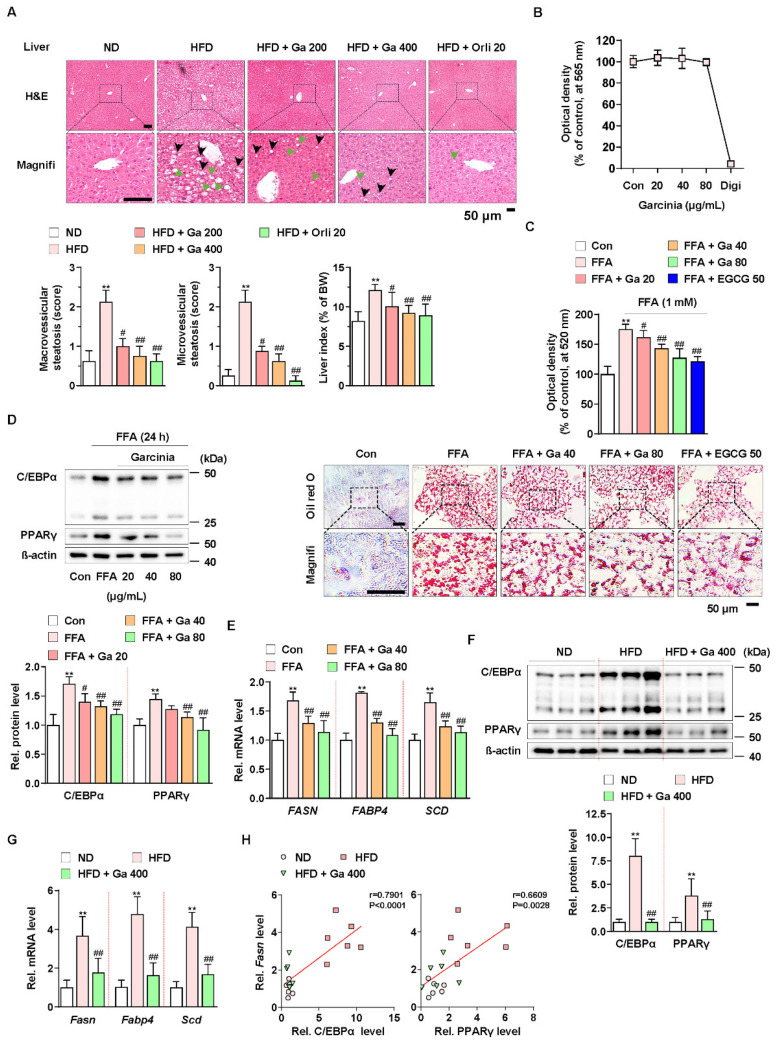
Anti-NALFD effect of *Garcinia cambogia* in HFD-fed mice reducing hepatic steatosis. (**A**) Representative images showing the effect of *G. cambogia* (Ga, 200 and 400 mg/kg) on HFD-induced hepatic steatosis. H&E staining of liver tissues was performed. The scores of macrovesicular (black arrow) and microvesicular (green arrow) steatosis and liver index were quantified, as described in the Methods section. Normal diet (ND)-fed mice were used as the negative control for high levels of fat accumulation, and orlistat (20 mg/kg) was used as the positive control for anti-obesity and anti-steatosis effects (*n* = 7 per group). Scale bars: 50 μm. Magnifi: magnification. ** *p* < 0.01 vs. ND-fed mice, ^#^ *p* < 0.05 and ^##^ *p* < 0.01 vs. HFD-fed mice. (**B**) MTT assay showing the effect of *G. cambogia* (20–80 μg/mL) on cell viability. HepG2 cells were treated with the indicated concentration of *G. cambogia* and digitonin (100 μg/mL, positive control) for 24 h (*n* = 5 per group). (**C**) oil red O assay and representative images showing the effect of *G. cambogia* (20–80 μg/mL) on free fatty acid (1 mM FFA)-induced lipid accumulation in HepG2 cells. FFA-induced HepG2 cells were treated with *G. cambogia* and EGCG (50 μM, positive control) for 24 h (*n* = 5 per group). Scale bar: 50 μm. Magnifi: magnification. ** *p* < 0.01 vs. Con, ^#^ *p* < 0.05 and ^##^ *p* < 0.01 vs. FFA. Effect of *G. cambogia* (20–80 μg/mL) on (**D**) C/EBPα and PPARγ expression and (**E**) transcript levels of *FASN*, *FABP4,* and *SCD* in FFA-treated HepG2 cells (d, *n* = 4 per group; e, *n* = 5 per group). Effect of *G. cambogia* on (**F**) C/EBPα and PPARγ expression and (**G**) *Fasn*, *Fabp4,* and *Scd* transcript levels of liver tissues from ND-fed, HFD-fed, and HFD-fed mice administered a high dose of *G. cambogia* (400 mg/kg) (*n* = 6 per group). (**H**) Correlations between *Fasn* transcript and C/EBPα and PPARγ protein levels in liver tissues (*n* = 6 per group). Each point represents one sample. Data are mean ± S.D.

**Figure 2:**
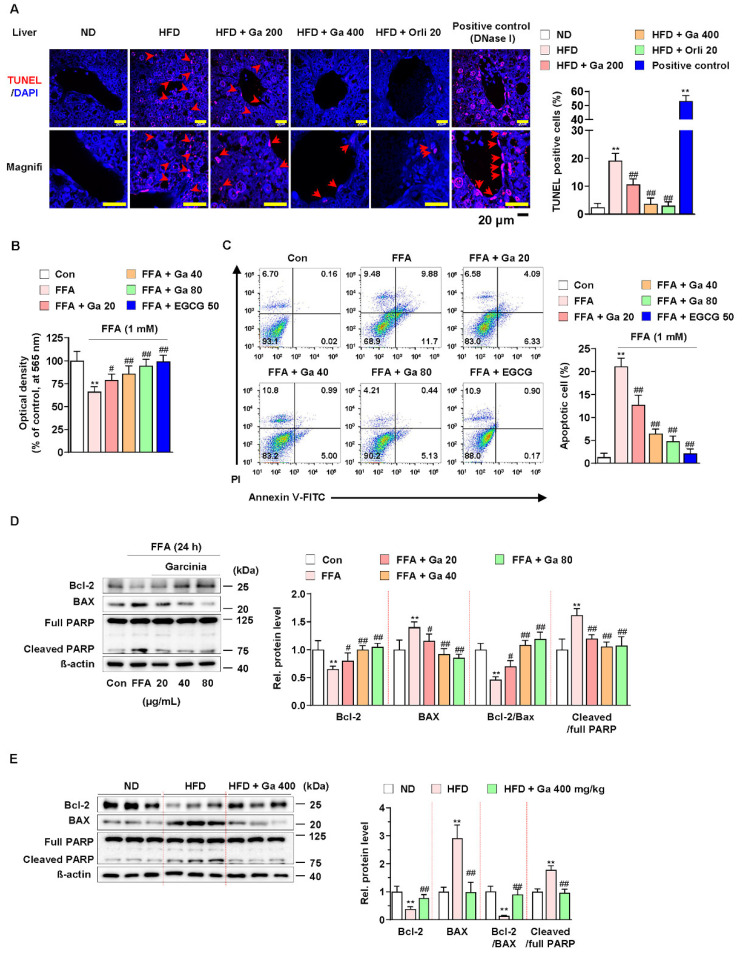
Effect of *Garcinia cambogia* on HFD-induced apoptosis in the liver. (**A**) Representative images of TUNEL staining and quantification data for the number of TUNEL-positive cells in HFD-induced liver tissues. Positive control: DNase I (30 U) treated group. Scale bar: 20 μm. (*n* = 6 per group). Magnifi: magnification. ** *p* < 0.01 vs. ND-fed mice, ^##^ *p* < 0.01 vs. HFD-fed mice. (**B**) MTT assay showing the effect of *G. cambogia* on FFA-induced cell viability. Cells were treated with *G. cambogia* (20–80 μg/mL) and EGCG (50 μM) for 24 h (*n* = 5 per group). ** *p* < 0.01 vs. Con, ^#^ *p* < 0.05 and ^##^ *p* < 0.01 vs. FFA. (**C**) Effect of *G. cambogia* on apoptosis in FFA-treated HepG2 cells. Cells were treated with *G. cambogia* (20–80 μg/mL) and EGCG (50 μM) for 24 h (*n* = 4 per group). The numbers of negatively and positively stained cells are expressed as percentages of the total number of cells. (**D**) Effect of *G. cambogia* on Bcl-2 and BAX expression and PARP cleavage in FFA-treated HepG2 cells (*n* = 4 per group). (**E**) Effect of *G. cambogia* on Bcl-2 and BAX expression and PARP cleavage of liver tissues from ND-fed, HFD-fed, and HFD-fed mice administered a high dose of *G. cambogia* (400 mg/kg) (*n* = 6 per group). Data are mean ± S.D.

All data were expressed as mean ± S.D. of 4–7 independent experiments mentioned in the respective figure legends or graphs. Statistical analyses were performed with GraphPad Prism software (Version 9, San Diego, CA, USA). The normality of the data distributions was tested using the Shapiro–Wilk test. A two-sided, unpaired Student’s *t*-test was used to analyze the difference between two groups of data. The data in Figures 1F and 2E were analyzed using Welch’s *t*-test for unequal variances. Differences across three or more groups were tested via one-way analysis of variance (ANOVA), followed by a post hoc analysis with Bonferroni’s test if F achieved statistical significance (*p* < 0.05) and there was no significant variance in homogeneity with the Barlett’s test. The correlation results were determined by Pearson’s rank test. Differences with *p* < 0.05 were considered statistically significant.

## 3. Results

### 3.1. Garcinia cambogia Attenuates NALFD in HFD-Induced Mice by Reducing Hepatic Steatosis

To investigate the effect of *G. cambogia* on NAFLD in HFD-fed mice, we administered *G. cambogia* and orlistat (positive control for the anti-obesity effect) for 8 weeks with HFD feeding. As shown in Table 1, *G. cambogia* significantly reduced weight gain resulting from HFD feeding, indicating an anti-obesity effect. In addition, administration of *G. cambogia* improved the abnormal serum levels of measured parameters, such as alanine aminotransferase (ALT) and aspartate aminotransferase (AST), markers of liver function, and triglycerides and total cholesterol, and markers of lipid metabolism, indicating that *G. cambogia* can regulate HFD-induced abnormal metabolic phenotype. Furthermore, *G. cambogia* inhibited the formation of hepatic lipid droplets and increased liver index, an indicator of NAFLD [46], resulting from HFD feeding (Figure 1A). There were no signs of obvious pathological changes in major organs such as the heart, spleen, lung, and kidney, implying no other organ toxicity (Figure S1).

To examine the underlying mechanism, we used the HepG2 cell line for an in vitro study. Compared to digitonin (100 μg/mL, positive control for cytotoxicity), *G. cambogia* (20–80 μg/mL) did not show any cytotoxic effects for 24 h in HepG2 cells (Figure 1B). In addition, *G. cambogia* significantly reduced free fatty acid (1 mM FFA, composed of oleate and palmitate)-induced lipid accumulation in HepG2 cells for 24 and 48 h, similar to EGCG (50 μM) as a known substance to have an anti-steatosis effect (Figure 1C and Figure S2A) [47].

Since hepatic lipid accumulation is regulated by lipogenesis-related molecules, we examined the levels of C/EBPα and PPARγ, two major transcription factors of lipogenesis, in FFA-treated HepG2 cells. As shown in Figure 1D, FFA treatment increased C/EBPα and PPARγ expression indicating de novo lipogenesis. However, *G. cambogia* significantly decreased FFA-induced alterations in the C/EBPα and PPARγ expression. Then we measured the effect of *G. cambogia* on the lipogenic gene transcription activated by C/EBPα and PPARγ. FFA treatment increased the levels of fatty acid synthase (*FASN*), fatty acid-binding protein 4 (*FABP4*), and stearoyl-CoA desaturase (*SCD*) transcripts, and *G. cambogia* significantly reduced the transcription of these genes (Figure 1E). As in the results of in vitro experiments, *G. cambogia* significantly reduced the protein levels of C/EBPα and PPARγ (Figure 1F) and the transcript levels of *Fasn*, *Fabp4,* and *Scd* (Figure 1G) in liver tissues of HFD-fed mice. As shown in Figure 1H, significant positive correlations were observed between *Fasn* transcript and C/EBPα and PPARγ proteins, indicating that increased C/EBPα and PPARγ by HFD were inhibited by *G. cambogia*, which regulated lipogenic genes such as *Fasn*. These results provide critical insight into the inhibitory effect of *G. cambogia* on NAFLD via suppressing hepatic steatosis.

### 3.2. Garcinia cambogia Suppressed FFA-Induced Apoptosis in the Liver

Excessive FFAs in the liver by consuming HFD cause apoptosis and triglyceride accumulation [48]. In addition, hepatocellular apoptosis plays a major role in the early mechanism underlying the progression of liver diseases, including NAFLD [49]. Thus, we examined whether *G. cambogia* regulates FFA-induced apoptosis. As shown in Figure 2A, *G. cambogia* administration significantly decreased the rate of HFD-induced apoptosis, as measured by TUNEL in liver tissues. In addition, FFA treatment reduced HepG2 cell viability; however, treatment with either *G. cambogia* (20–80 μg/mL) or EGCG (50 μM) improved cell viability, which had been reduced by FFA for 24 and 48 h (Figure 2B and Figure S2B). Moreover, *G. cambogia* and EGCG (positive control of anti-apoptosis effect [50]) inhibited FFA-induced cell apoptosis, indicating that the improvement of cell viability by *G. cambogia* was due, at least in part, to suppression of apoptosis (Figure 2C).

Since apoptosis is regulated by proapoptotic and antiapoptotic molecules such as Bcl-2 family members related to mitochondrial dysfunction and subsequent caspase-3 activation and cleavage of PARP [51,52,53], we examined the effect of *G. cambogia* on the alteration of apoptosis-related molecules in FFA-treated HepG2 cells. FFA treatment significantly decreased the expression of Bcl2 apoptosis regulator (Bcl-2) and increased the expression of Bcl-2-associated X protein (BAX), thereby suppressing the Bcl-2/BAX ratio, and increased the levels of caspase-3 activity and cleaved PARP indicating the induction of apoptosis (Figure S3A and Figure 2D). These results were similar in experiments observed in the liver tissues of HFD-fed mice (Figure S3B and Figure 2E). In both in vitro and in vivo experiments, *G. cambogia* (20–80 μg/mL) treatment increased the Bcl-2/Bax ratio and inhibited caspase-3 activation and PARP cleavage, indicating that *G. cambogia* suppresses apoptosis-related apoptosis pathways. Taken together, these results suggest that *G. cambogia* alleviates HFD- and FFA-induced apoptosis to inhibit the progression of NAFLD.

### 3.3. Garcinia cambogia Inhibits HFD- and FFA-Induced ROS Production via NRF2 Activation

**Figure 3:**
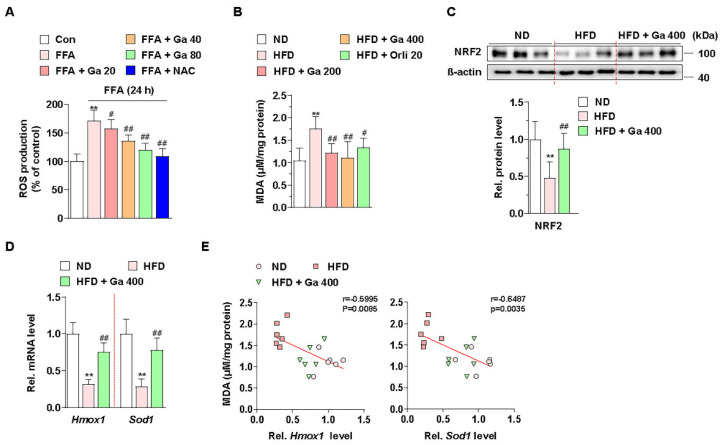
Effect of *Garcinia cambogia* on HFD- and FFA-induced ROS production, and NRF2 and downstream gene activation. (**A**) Effect of *G. cambogia* on ROS level in FFA-treated HepG2 cells. Cells were treated with *G. cambogia* (20–80 μg/mL) or NAC (5 mM, positive control of antioxidant) for 24 h. ROS production was measured using H_2_DCFDA (*n* = 5 per group). ** *p* < 0.01 vs. Con, ^#^ *p* < 0.05 and ^##^ *p* < 0.01 vs. FFA. (**B**) Effect of *G. cambogia* on malondialdehyde (MDA, indicating the intensity of lipid peroxidation) level of serum in HFD-fed mice (*n* = 7 per group). Effect of *G. cambogia* on (**C**) NRF2 expression and (**D**) *Hmox1* and *Sod1* transcript levels of liver tissues from ND-fed, HFD-fed, and HFD-fed mice administered a high dose of *G. cambogia* (400 mg/kg) (*n* = 6 per group). (**E**) Correlations between MDA level and *Hmox1* and *Sod1* transcript levels in liver tissues (*n* = 6 per group). Each point represents one sample. ** *p* < 0.01 vs. ND-fed mice, ^#^ *p* < 0.05 and ^##^ *p* < 0.01 vs. HFD-fed mice. Data are mean ± S.D.

Excessive accumulation of FFAs by energy imbalance in the liver produces ROS, and high levels of ROS can induce lipid accumulation via failure of lipid metabolism and liver damage via apoptosis [48]. Thus, we examined whether *G. cambogia* regulates FFA-induced ROS production. Intracellular ROS levels were increased by FFA in HepG2 cells, and both *G. cambogia* (20–80 μg/mL) and NAC (5 mM, positive control for the antioxidant effect [54]) significantly reduced FFA-induced ROS production for 24 and 48 h (Figure 3A and Figure S2C). In addition, the serum level of MDA, as a marker of oxidative stress and antioxidant status [55,56], was increased in HFD-fed mice, but *G. cambogia* administration alleviated the increase in the MDA level, indicating *G. cambogia*-mediated antioxidant effect (Figure 3B).

Cellular ROS are regulated by endogenous antioxidant defense systems such as enzymatic antioxidants like heme oxygenase 1 (HO-1) and superoxide dismutase (SOD) [57]. In addition, NRF2, one of the major transcription factors of the antioxidant defense system, regulates *HMOX1* and *SOD* transcription for ROS homeostasis [58]. Thus, we examined whether *G. cambogia* regulates NRF2 levels in HFD-fed mice and FFA-treated HepG2 cells. As a result of measuring NRF2 expression level in the livers of HFD-fed mice, we found that *G. cambogia* reduced HFD-induced inhibition of NRF2 expression (Figure 3C). In addition, *Hmox1* and *Sod1* gene expression, which had been inhibited by HFD, were recovered by *G. cambogia* (Figure 3D). Furthermore, significant negative correlations between MDA protein and *Hmox1* and *Sod1* transcripts were observed, indicating that increased MDA by HFD was inhibited by *G. cambogia*-induced alterations of the antioxidant defense system (Figure 3E). Taken together, *G. cambogia* activates the intracellular antioxidant defense system by increasing the expressions of NRF2 and downstream genes to reduce ROS production.

### 3.4. Increased NRF2 Nuclear Expression and ARE Activation by Garcinia cambogia Regulates Antioxidant Gene Expression in FFA-Treated HepG2 Cells

**Figure 4:**
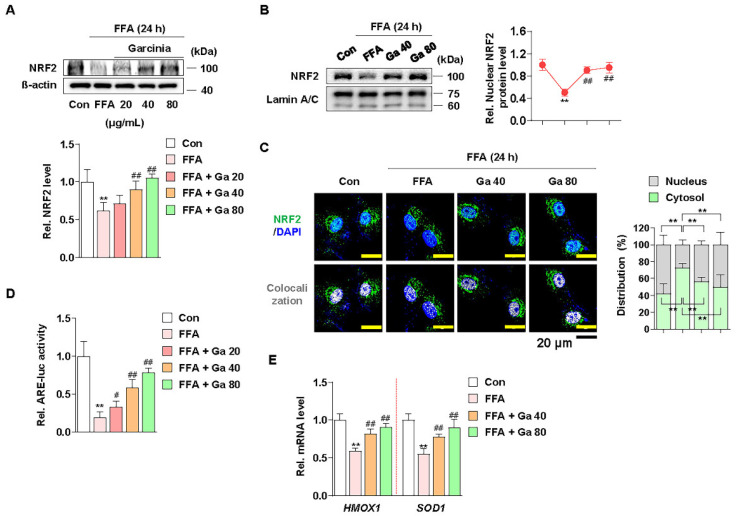
Effect of *Garcinia cambogia* on NRF2-ARE activation in FFA-induced HepG2 cells. (**A**) Effect of *G. cambogia* (20–80 μg/mL) on NRF2 expression in FFA-treated HepG2 cells (*n* = 4 per group). (**B**) Effect of *G. cambogia* on NRF2 nuclear expression in FFA-treated HepG2 cells. Cells were treated with *G. cambogia* (40 and 80 μg/mL) for 12 h, and cells were fractionated into nucleus compartment as described in the Methods section (*n* = 4 per group). (**C**) Representative immunofluorescence images of NRF2 in FFA-treated HepG2 cells. Colocalized FITC (i.e., NRF2) and DAPI (i.e., nuclei) were quantified using ImageJ software, and relative intensities in the nuclear and cytoplasmic fractions were expressed as a histogram. Scale bars: 20 μm (*n* = 5 per group). (**D**) Effect of *G. cambogia* on ARE promoter activity in FFA-treated HepG2 cells. ARE-luc vector-transfected HepG2 cells were cotreated FFA and *G. cambogia* (20–80 μg/mL) for 12 h and detected ARE promoter activity (*n* = 6 per group). (**E**) Effect of *G. cambogia* (20–80 μg/mL) on the transcript levels of *HMOX1* and *SOD1* in FFA-treated HepG2 cells (*n* = 5 per group). ** *p* < 0.01 vs. Con, ^#^ *p* < 0.05 and ^##^ *p* < 0.01 vs. FFA. Data are mean ± S.D.

NRF2 functions as a transcription factor in the nucleus by ARE promoter activation and subsequent modulation of antioxidant gene expression [59]. Thus, we examined whether *G. cambogia* affected the nuclear expression of NRF2 and ARE promoter activity and in FFA-treated HepG2 cells. Similar to the in vivo results, FFA significantly reduced the expression of NRF2, and *G. cambogia* restored FFA-induced inhibition of NRF2 expression (Figure 4A). Moreover, *G. cambogia* increased the expression of NRF2 in the nucleus (Figure 4B,C), which could influence the expression of downstream genes. In addition, *G. cambogia* increased FFA-induced reduction of ARE activity (Figure 4D). As expected, *G. cambogia* restored FFA-induced reduction of *HMOX1* and *SOD1* gene expressions (Figure 4E). Taken together, these results suggest that *G. cambogia* activates the NRF2-ARE signaling pathway to inhibit FFA-induced abnormal ROS production.

### 3.5. Hydroxycitric Acid Contributed to Suppress FFA-Induced ROS Production, Lipid Accumulation, and Apoptosis

**Figure 5:**
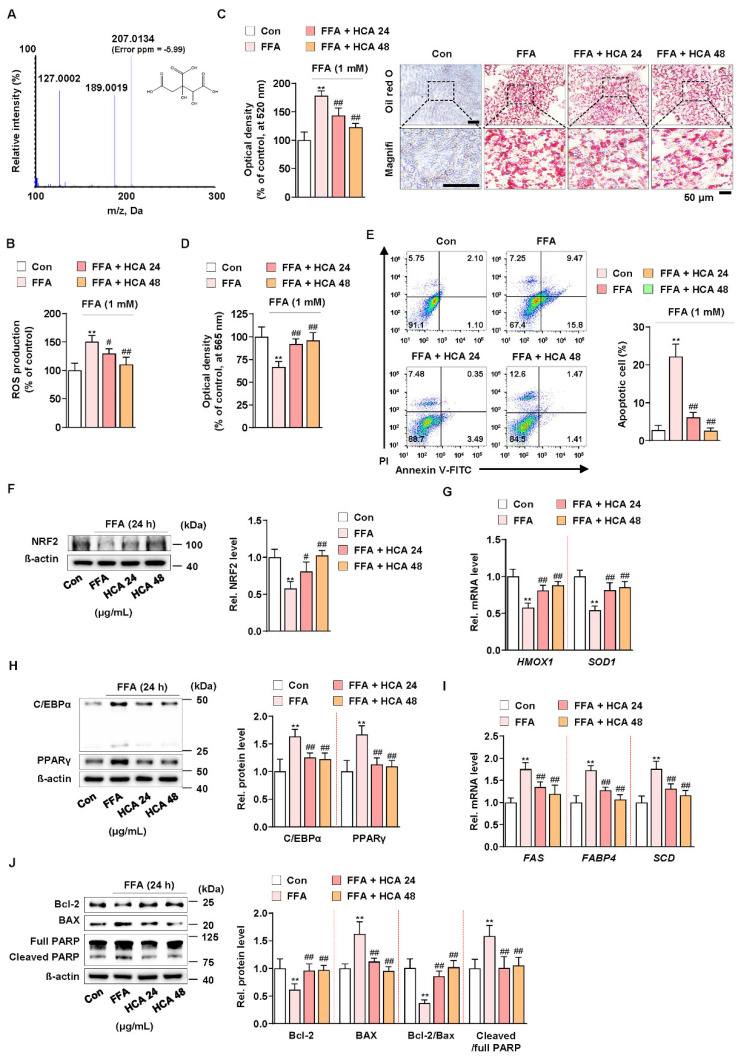
Effect of hydroxycitric acid on FFA-induced ROS production, lipid accumulation, and apoptosis in HepG2 cells. (**A**) MS/MS spectrum of hydroxycitric acid [M − H]^−^. The parent ion was the deprotonated [M − H]^−^ ion at *m*/*z* 207.0 and the most abundant ion in product ion scan mode. (**B**) ROS measurement using H_2_DCFDA in FFA-treated HepG2 cells treated with HCA (24 and 48 μg/mL) (*n* = 5 per group). (**C**) Oil red O assay and representative images showing the effect of HCA (24 and 48 μg/mL) on FFA-induced lipid accumulation in HepG2 cells. Cells were treated with HCA (24 and 48 μg/mL) for 24 h (*n* = 5 per group). Scale bar: 50 μm. Magnifi: magnification. (**D**) MTT assay showing the effect of HCA (24 and 48 μg/mL) on FFA-induced cell viability. Cells were treated with HCA (24 and 48 μg/mL) for 24 h (*n* = 5 per group). (**E**) Effect of HCA (24 and 48 μg/mL) on apoptosis in FFA-treated HepG2 cells. Cells were treated with HCA (24 and 48 μg/mL) for 24 h (*n* = 4 per group). The numbers of negatively and positively stained cells are expressed as percentages of the total number of cells. Effect of HCA (24 and 48 μg/mL) on the levels of (**F**) NRF2 expression, (**G**) *HMOX1* and *SOD1* transcriptions, (**H**) C/EBPα and PPARγ expression, (**I**) *FAS, FABP4* and *SCD* transcriptions, and (**J**) Bcl-2 and BAX expression and PARP cleavage in FFA-treated HepG2 cells (*n* = 4 per group in f, h, and j; *n* = 5 per group in **G**,**I**). ** *p* < 0.01 vs. Con, ^#^ *p* < 0.05 and ^##^ *p* < 0.01 vs. FFA. Data are mean ± S.D.

A recent study reported that hydroxycitric acid (HCA) reduced oleic acid-induced steatosis and oxidative stress in primary chicken hepatocytes [60]. In addition, it is known that *G. cambogia* contains from 20 to 60% hydroxycitric acid (HCA) as a main component [24]. The HCA content of *G. cambogia* used in this experiment was analyzed using liquid chromatography-high resolution mass spectrometry (LC-HRMS), and HCA content was 59.55 ± 2.66% (Figure 5A). To verify whether the same action of *G. cambogia* as shown so far is actually the same in HCA, the effect of HCA (24 and 48 μg/mL) at concentrations equivalent to those of *G. cambogia* (40 and 80 μg/mL) on FFA-induced ROS production, lipid accumulation, and apoptosis was examined in FFA-treated HepG2 cells. HCA significantly inhibited FFA-induced ROS production and lipid accumulation (Figure 5B,C). In addition, HCA attenuated FFA-induced reduction of cell viability and induction of apoptosis (Figure 5D,E). Similar to the effect of *G. cambogia*, HCA ameliorated FFA-induced decrease of NRF2 expression and *HMOX1* and *SOD1* transcription, indicating an antioxidant effect of HCA (Figure 5F,G). Furthermore, HCA regulated lipogenesis- and apoptosis-related signaling pathways. As shown in Figure 5H, HCA inhibited FFA-induced upregulation of C/EBPα and PPARγ expression, *FAS*, *FABP4,* and *SCD* transcription to suppress lipogenesis (Figure 5H,I). In addition, HCA significantly altered the FFA-induced Bcl-2/BAX ratio, caspase-3 activity, and PARP cleavage to reduce apoptosis (Figure 5J and Figure S3C). These results indicated that the underlying effects of *G. cambogia* are due, at least in part, to HCA.

**Figure 6:**
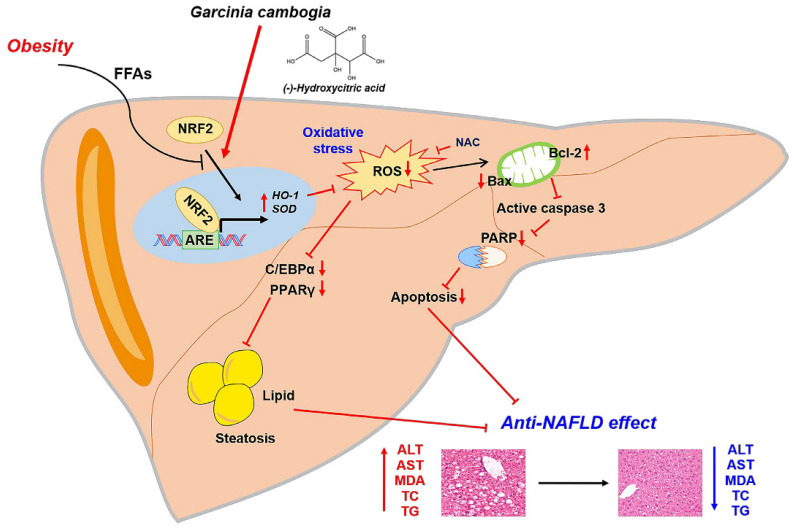
Proposed mechanism of the protective effect of *G. cambogia* against NAFLD. *G. cambogia* inhibited HFD-induced NAFLD by activating the NRF2-ARE-mediated antioxidant defense system, leading to inhibition of hepatic steatosis and apoptosis in the liver.

Taken together, our results support the conclusion that NRF2 activation by *G. cambogia* and HCA contributed to the antioxidant effect also affects NAFLD progression through hepatic steatosis and apoptosis inhibition (Figure 6).

## 4. Discussion

To date, there are no effective pharmacological therapies for NAFLD; thus, therapeutic approaches to manage NAFLD by dietary supplementation, for example, silymarin [61], berberine [19], and resveratrol [62], are considered. Although *G. cambogia*, a natural product used as a weight-loss supplement in many countries, has been reported to have an anti-NAFLD effect [28], the underlying mechanism is poorly understood.

In the present study, we demonstrated for the first time that (1) *G. cambogia* suppressed hepatic steatosis and apoptosis in the liver of HFD-fed mice and in FFA-treated HepG2 cells; (2) *G. cambogia* inhibited HFD- and FFA-induced upregulation of C/EBPα and PPARγ expression, and *FASN*, *FABP4,* and *SCD* transcription to regulate lipogenesis; (3) *G. cambogia* reduced the HFD- and FFA-induced alterations of the Bcl-2/BAX ratio and PARP cleavage to regulate apoptosis; and (4) these normalizations were due to the antioxidant effect of *G. cambogia* and HCA through NRF2-ARE pathway activation. These findings are summarized in Figure 6.

The effect of *G. cambogia* and HCA, its main constituent, on lipogenesis has been reported in several studies in animal models [24,25,63]. It was recently published that the regulatory effect of HCA on lipid accumulation in cultured primary chicken hepatocytes reduces the acetyl-CoA supply, which is mainly achieved via inhibition of ATP-citrate lyase and acceleration of energy metabolism [63]. In addition, HCA reduced steatosis and oxidative stress by regulating the AMPK-mediated signaling pathway [60]. However, our study focused on the antioxidant effect of *G. cambogia* and HCA via NRF2-ARE activation, and it modulated lipogenesis and apoptosis to ameliorate NAFLD.

Reactive oxygen species (ROS) are generated in cells by several factors, such as stressor responses and environmental factors [64]. Excessive accumulation of free fatty acids (FFAs) due to western diets, including high-fat, is one of the important factors. ROS overproduction induces lipid peroxidation, mitochondrial dysfunction, and lipogenic protein activation to increase fat accumulation in the liver [12]. In addition, excessive ROS activates hepatic C/EBPα and PPARγ, key transcription factors that control many lipogenic genes, such as *FASN*, *FABP4,* and *SCD*, to promote NAFLD progression [65,66]. Our study firstly revealed that *G. cambogia* and HCA inhibited HFD- and FFA-induced C/EBPα and PPARγ activation by reducing intracellular ROS levels and leading to suppression of lipogenic gene transcription and hepatic steatosis (Figure 1, Figure 3A,B and Figure 5).

Increased ROS levels have been indicated to enhance the expression of apoptosis-related proteins [48]. In addition, apoptosis is a prominent feature of NAFLD progression and liver disease [67]. Since ROS causes mitochondrial dysfunction and alterations in Bcl-2 family proteins such as Bcl-2 and BAX, which are major regulators of apoptotic processes and lead to collapse of the mitochondrial membrane potential [68], we examined the effect of *G. cambogia* on Bcl-2 and BAX expression and PARP cleavage. This study revealed that *G. cambogia* and HCA significantly attenuated the HFD- and FFA-induced decrease in the Bcl-2/BAX ratio and induction of PARP cleavage, indicating suppression of ROS-mediated apoptosis (Figure 2 and Figure 5E,J).

*G. cambogia* contains many components, including alkaloids, flavonoids, phenolic compounds, saponins, tannins, carbohydrates, and proteins [24]. To date, few single compounds have been isolated from *G. cambogia*. HCA, garcinol, and guttiferone K isolated from the extract showed protective antioxidant effects against lipid and protein oxidation/glycation and endoplasmic reticulum (ER) stress [69,70,71]. In addition, advanced glycation end products (AGE) can be a potential biomarker of the NAFLD stage since AGE showed good criteria for dividing patients with minimal steatosis (BARD score 0–1) vs. moderate steatosis (BARD score 2–4) [72]. Furthermore, a high intake of fat and carbohydrates can induce ER stress, and dysfunction of ER develops metabolically-driven NAFLD progress [73,74]. Although this study revealed that HCA had therapeutic effects by suppressing lipid accumulation and apoptosis in liver cells, other components may be effective to NAFLD since recent clinical trials suggest that protein oxidation/glycation and ER stress are involved in liver fibrosis in NAFLD [75,76]. Thus, further studies are needed to investigate additional anti-NAFLD effects and the underlying mechanism of the *G. cambogia* and its components.

Finally, it has been reported that *G. cambogia* is associated with hepatotoxicity, including lipid peroxidation and oxidative stress genes [77]. However, this assertion is controversial because our study showed that *G. cambogia* administration suppressed the HFD-induced increases in ALT and AST levels (Table 1), and *G. cambogia* inhibited HFD- and FFA-induced oxidative stress via upregulating antioxidative barriers. In addition, HCA, the main constituent, does not promote liver toxicity or inflammation [78].

## 5. Conclusions

In conclusion, this study demonstrated that *G. cambogia* inhibits HFD-induced NAFLD by suppressing hepatic steatosis and apoptosis through the regulation of lipogenesis- and apoptosis-related signaling pathways. These effects are due to the antioxidant effect of *G. cambogia* and HCA by activating the NRF2-ARE pathway. Our findings provide new insight into the mechanism by which *G. cambogia* suppresses NAFLD progression.

## Figures and Tables

**Table 1:** Body weight and biochemical markers of animal models (*n* = 7 per group).

Body Weight (g)	ND	HFD	HFD + Ga 200	HFD + Ga 400	HFD + Orli 20
Body weight (g, initial time)	22.29 ± 0.92	23.51 ± 1.09	23.27 ± 0.73	22.89 ± 1.08	22.57 ± 0.76
Body weight (g, finish time)	27.56 ± 1.38	34.07 ± 1.37 **	31.54 ± 2.20	29.27 ± 0.70 ^##^	28.81 ± 1.96 ^##^
Weight gain	5.27 ± 1.48	10.56 ± 1.31 **	8.27 ± 2.38	6.39 ± 1.04 ^##^	6.24 ± 1.29 ^##^
ALT (U/I)	53.00 ± 7.14	84.69 ± 21.61 **	63.33 ± 8.76 ^#^	53.33 ± 6.83 ^##^	54.79 ± 7.76 ^##^
AST (U/I)	73.50 ± 6.82	100.2 ± 10.97 **	82.50 ± 11.29 ^#^	75.83 ± 5.85 ^##^	77.83 ± 4.02 ^##^
Triglyceride (mg/dL)	86.42 ± 38.00	215.2 ± 30.96 **	130.0 ± 33.76 ^##^	113.3 ± 19.66 ^##^	173.7 ± 42.58
Total cholesterol (mg/dL)	140.0 ± 7.07	240.2 ± 25.93 **	201.7 ± 14.38 ^#^	180.8 ± 14.63 ^##^	168.0 ± 27.28 ^##^

** *p* < 0.01 vs. ND, ^#^ *p* < 0.05 and ^##^ *p* < 0.01 vs. HFD. Data are mean ± S.D.

## Data Availability

Data is contained within the article or supplementary material.

## Supplementary Materials

The following are available online at www.mdpi.com/article/10.3390/antiox10081226/s1, Figure S1: *G. cambogia* attenuates the HFD-induced NAFLD without organ toxicity, Figure S2: Effect of *Garcinia cambogia* on FFA-induced hepatic steatosis, cell viability and ROS production in HepG2 cells for 48 h, Figure S3: Effect of *Garcinia cambogia* and HCA on HFD- and FFA-induced caspase 3 activation, Table S1: Primers for real-time PCR.
