# Supplementary material for: Garcinia cambogia Ameliorates Non-Alcoholic Fatty Liver Disease by Inhibiting Oxidative Stress-Mediated Steatosis and Apoptosis through NRF2-ARE Activation
Source: Antioxidants (Basel). 2021 Jul 29;10(8):1226. doi: 10.3390/antiox10081226 (PMC8388869; doi:10.3390/antiox10081226)
Supplement: Supplementary file 1 [file antioxidants-10-01226-s001.zip › antioxidants-1304020-supplementary.pdf]

antioxidants

MDPI

Article

Garcinia cambogia Ameliorates Non-Alcoholic Fatty Liver Disease by Inhibiting Oxidative Stress-Mediated Steatosis and Apoptosis through NRF2-ARE Activation

Joo-Hui Han [1,*], Min-Ho Park [2] and Chang-Seon Myung [1,*]

[1] Department of Pharmacology, College of Pharmacy, Chungnam National University, Daejeon 34134, Korea
[2] Institute of Drug Research and Development, College of Pharmacy, Chungnam National University, Daejeon 34134, Republic of Korea
* Correspondence: han5621@cnu.ac.kr (J.-H.H.); cm8r@cnu.ac.kr (C.-S.M.); Tel.: +82-42-821-7299 (J.-H.H.); +82-42-821-5923 (C.-S.M.)

Supporting Information

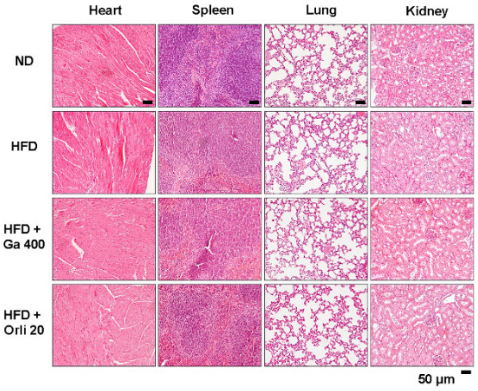

Figure S1. *G. cambogia* attenuates the HFD-induced NAFLD without organ toxicity. Histological images of the heart, spleen, lung and kidney using H&E staining. Scale bars: 50 μm.

Citation: Han, J.-H.; Park, M.-H.; Myung, C.-S. *Garcinia cambogia* Ameliorates Non-Alcoholic Fatty Liver Disease by Inhibiting Oxidative Stress-Mediated Steatosis and Apoptosis through NRF2-ARE Activation. *Antioxidants* **2021**, *10*, x. https://doi.org/10.3390/antiox10081226

Academic Editor: Greg Barritt

Received: 13 July 2021
Accepted: 28 July 2021
Published: 29 July 2021

Publisher's Note: MDPI stays neutral with regard to jurisdictional claims in published maps and institutional affiliations.

Copyright: © 2021 by the authors. Submitted for possible open access publication under the terms and conditions of the Creative Commons Attribution (CC BY) license (http://creativecommons.org/licenses/by/4.0/).

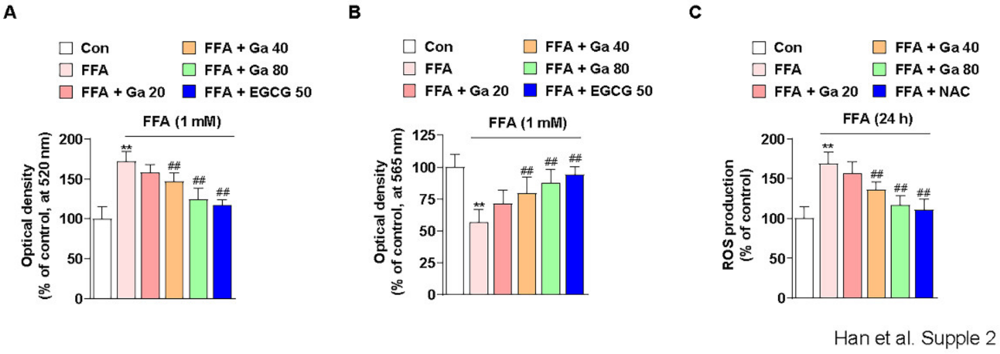

Figure S2. Effect of *Garcinia cambogia* on FFA-induced hepatic steatosis, cell viability and ROS production in HepG2 cells for 48 h. (a) Oil red O assay showing the effect of *G. cambogia* (20-80 μg/mL) on free fatty acid (1 mM FFA)-induced lipid accumulation in HepG2 cells. FFA-induced HepG2 cells were treated with *G. cambogia* and EGCG (50 μM, positive control) for 48 h (n=5 per group). (b) MTT assay showing the effect of *G. cambogia* on FFA-induced cell viability. Cells were treated with *G. cambogia* (20-80 μg/mL) and EGCG (50 μM) for 48 h (n=5 per group). (c) Effect of *G. cambogia* on ROS level in FFA-treated HepG2 cells. Cells were treated with *G. cambogia* (20-80 μg/mL) or NAC (5 mM, positive control of antioxidant) for 48 h. ROS production was measured using H_2DCFDA (n=5 per group). $^{**}p < 0.01$ vs. Con, $^{\#\#}p < 0.01$ vs. FFA.

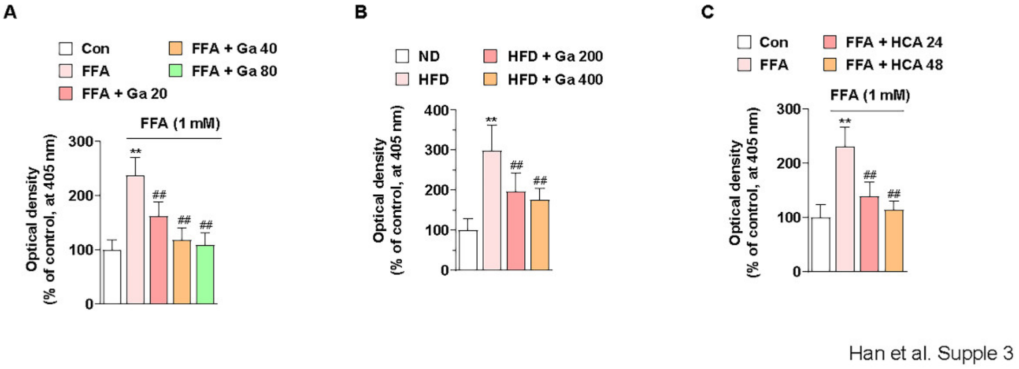

Figure S3. Effect of *Garcinia cambogia* and HCA on HFD- and FFA-induced caspase 3 activation. (a) Caspase 3 assay showing the effect of *G. cambogia* (20-80 μg/mL) on FFA (1 mM)-induced caspase 3 activation in HepG2 cells. FFA-induced HepG2 cells were treated with *G. cambogia* for 24 h (n=5 per group). (b) Effect of *G. cambogia* on caspase 3 activity of liver tissues in HFD-fed mice (n=6 per group).
(c) Caspase 3 assay showing the effect of HCA (24 and 48 μg/mL) on FFA (1 mM)-induced caspase 3 activation in HepG2 cells. FFA-induced HepG2 cells were treated with HCA for 24 h (n=5 per group).

Table S1. Primers for real-time PCR

Genes	Accession number	Sense primers (5`-3`)	Antisense primers (5`-3`)
Mouse/Human *β-actin*	NM_001101.5/ NM_031144.3	TCCATCATGAAGTGTGACGT	GCTCAGGAGGAG-CAATGAT
Human *FASN*	NM_004104.5	CCCCTGATGAA-GAAGGATCA	ACTCCACAGGTGG-GAACAAG

Human *FABP4*	NM_001442.3	ACAGGAAAGTCAAGAG-CACC	AACTCTCGTG-GAAGTGACGC
Human *SCD*	NM_005063.5	TGATGTTCCAGAG-GAGGTACT	AGCACCACAGCATATCG-CAA
Human *HMOX1*	NM_002133.3	CTGCTCAACATCCAGCTCTTTG	ATCTTGCACTTTGTT-GCTGGC
Human *SOD1*	NM_000454.5	GTGAAGGTGTGGGGAA-GCAT	AAGTCTCCAACATGCCTCTCTT
Mouse *Fasn*	NM_007988.3	CGGTG-TATCCTGCTGTCCAA	TGGGCTT-GTCCTGCTCTAAC
Mouse *Fabp4*	NM_024406.2	AAATCACCGCAGAC-GACAGG	CATAACACATTCCACCAC-CAGC
Mouse *Scd*	NM_009127.4	GCCCACATGCTCCAAGAG	GAGGGGCACCGTCTTCAC
Mouse *Hmox1*	NM_010442.2	CCCCAC-CAAGTTCAAACAGC	GCTCCTCAAACAGCTCAATGT
Mouse *Sod1*	NM_011434.2	CGATGAAAGCGGTGTGCG	TGCACTGGTACAGCCTT-GTGT
